# Temporal frequency dependence of the polarity inversion between upper and lower visual field in the pattern-onset steady-state visual evoked potential

**DOI:** 10.1007/s10633-022-09904-9

**Published:** 2022-10-22

**Authors:** Roman Kessler, Sven P. Heinrich

**Affiliations:** 1 Department of Neuropsychology, Max Planck Institute for Human Cognitive and Brain Sciences, Leipzig, Germany; 2 Eye Center, Faculty of Medicine, Medical Center – University of Freiburg, Freiburg, Germany

**Keywords:** VEP, SSVEP, Polarity inversion, Polarity reversal, Cruciform model

## Abstract

**Purpose:**

According to the cruciform model, the upper and lower halves of the visual field representation in the primary visual cortex are located mainly on the opposite sides of the calcarine sulcus. Such a shape would have consequences for the surface-recorded visual evoked potential (VEP), as V1 responses to stimulation of the upper and lower hemifield manifest with opposite polarity (i.e., polarity inversion). However, the steady-state VEP results from a complex superposition of response components from different cortical sources, which can obscure the inversion of polarity. The present study assesses the issue for different stimulation frequencies which result in different patterns of superposition in the steady-state response.

**Methods:**

Sequences of brief pattern-onset stimuli were presented at different stimulation rates ranging from 2 Hz (transient VEP) to 13 Hz (steady-state VEP). The upper and lower hemifields were tested separately and simultaneously. The data were assessed both in the time domain and in the frequency domain.

**Results:**

Comparing the responses to the stimulation of upper and lower hemifield, polarity inversion was present within a limited time interval following individual stimulus onsets. With increasing frequency, this resulted in an approximate inversion of the full steady-state response and consequently in a phase shift of approximately 180° in the time-domain response. Polarity inversion was more prominent at electrode Pz, also for transient responses. Our data also demonstrated that the sum of the hemifield responses is a good approximation of the full-field response.

**Conclusion:**

While the basic phenomenon of polarity inversion occurs irrespective of the stimulus frequency, its relative impact on the steady-state response as a whole is the largest for high stimulation rates. We propose that this is because longer-lasting response components from other visual areas are not well represented in the steady-state VEP at higher frequencies.

**Supplementary Information:**

The online version contains supplementary material available at 10.1007/s10633-022-09904-9.

## Introduction

Steady-state visual evoked potentials (ssVEPs) have a number of advantages over their transient counterparts. For instance, the high stimulation rate usually results in a better signal-to-noise ratio for a given recording duration [1], and frequency domain analysis facilitates objective response detection and statistical assessment [2–7]. This is of particular relevance in applications such as objective acuity estimation [8], where testing at multiple spatial frequencies lengthens recording time and a core part of analysis is to decide the presence of a response.

**Fig. 1:**
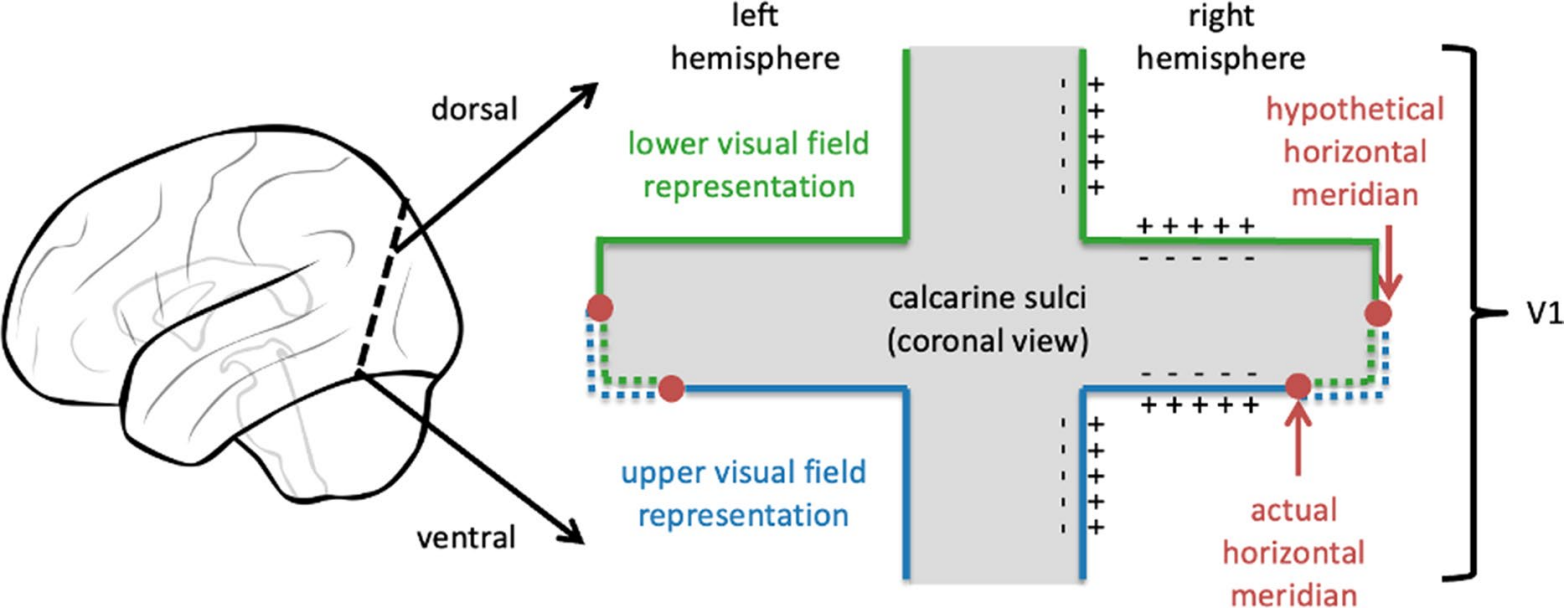
Schematic representation of the cruciform model, illustrating the hypothetical arrangement of primary visual cortices in bilateral calcarine sulcus. The slice on the right side corresponds to a tilted coronal cut within the occipital cortex, as indicated on the scheme on the left side. The lower visual field is represented on dorsal parts of V1, whereas the upper visual field is represented on ventral parts of V1. Importantly, major parts of the upper and lower visual hemifields lie opposite to each other on the side flanks of the hypothetical cruciform, leading to opposite electrophysiological polarity when activated. The horizontal meridian is on the ventral flank of the cruciform rather than on its apex, but subject to inter-individual variability. Consequently, polarity of signals from V1 during full visual field stimulation within a certain eccentricity should have tendencies to exhibit polarity rather like lower visual field stimulation than upper visual field stimulation

An important part of the pattern-onset response, including the C1 peak, originates from the primary visual cortex, which is located in a retinotopically organized fashion, in the calcarine sulcus [9]. Central parts of the visual field are processed in the most posterior part of the primary visual cortex. With increasing stimulus eccentricity, processing shifts to more anterior parts [10]. Furthermore, the upper and lower visual field are represented on opposite sides of the calcarine sulcus [10] (Fig. 1).

The anatomical layout of the primary visual cortex has consequences for electrophysiological measurements, as the difference in surface potential that is measured with the electroencephalogram (EEG) is mostly driven by directional currents that are generated by the pyramidal cells in layers III, V and VI of neocortex [11]. The pyramidal cells are mostly oriented perpendicularly to the cortical surface, which means that the measured signal polarity heavily depends on the geometry of the cortical surface [11]. According to the cruciform model, the calcarine sulcus is shaped in a cruciform manner with the primary visual cortex embedded such that the upper and lower hemifields’ representations lie largely on opposite sides of the sulcus [10] (Fig. 1). Thus, stimulation of upper and lower visual hemifields leads to opposite polarities measured at the scalp. This has been confirmed by several visual evoked potential (VEP) studies [9, 10] [12–14] and can nicely be seen in multifocal VEP recordings [15, 16]. In light of the cruciform model, the degree to which the opposite polarity (i.e., polarity inversion) occurs depends on the electrode configuration and the representation of the horizontal meridian within the calcarine sulcus, which is typically shifted somewhat toward the sulcus’ ventral flank [13] (Fig. 1).

When presenting single events of pattern onset stimuli, a polarity inversion between upper and lower hemifield stimulation is reported for certain components. The first of these is the C1 component, occurring with an onset latency of 40–70 ms and a peak latency of 60–100 ms [9]. The second component is C2, peaking at 130 ms with opposite polarity to C1, but also reported to show polarity inversion between hemifields [17]. C1 and probably C2 are thought to originate mainly from primary visual cortex [9, 17–19], and in part of secondary visual areas V2 and V3 [18–21]. Whereas it is assumed that C1 reflects an initial cortical volley originating from LGN, C2 is thought to represent feedback activity from higher visual areas such as hMT + and is observed when pattern reversal stimuli have been used for stimulation [17]. Other early components, such as P1, do not demonstrate polarity inversion [17].

Regarding steady-state VEPs, preliminary evidence is mixed. Elgohary & Heinrich [22] noted that changes in polarity were inconsistent between participants in a study that assessed hemifield stimulation resulting from incorrect stimulus fixation. Horn et al. [23] did not address the issue, but in a figure showing steady-state pattern-reversal multifocal VEPs, only some steady-state traces exhibited a polarity inversion. The interpretation is complicated by the fact that not all traces in the figure originated from the same electrode pair.

However, a body of studies contradicts the cruciform model. Some conclude that the calcarine fissure is not part of the VEP source generators [24, 25], whereas others conclude otherwise [26]. Some studies did not find polarity inversion [25]. Other studies, however, propose that different dynamics in processing between the upper and lower visual field—possibly associated with different relevance of information from both hemifields—might be responsible for the pattern which we label polarity inversion, rather than the proposed architecture of the cruciform model [27]. For the purpose of the present study, we use the cruciform hypothesis as a working model while acknowledging the contradictory nature of the available literature. Although the cruciform model represents only one way to interpret results, assumptions in this respect do not change the results of the experiments themselves, in which we aim to represent the behavior of electrophysiological responses to stimulation in different hemifields and at different frequencies.

Because the steady-state VEP results from a superposition of multiple response components that would be temporally distinct in the transient VEP [28, 29], the situation of polarity is relatively complex and likely to depend on the stimulation frequency. The present study was conducted to assess this issue in more detail and characterize the polarity inversion at different stimulation frequencies. We hypothesized that polarity inversion would be presented with some stimulation frequencies but possibly not with others, for instance due to frequency-dependent constructive and destructive superposition of the constituent response components that fuse into the steady-state signal. Of these constituent components, some may show more polarity inversion than others. To test this hypothesis, we presented pattern onset checkerboard stimuli in either the upper, lower or both hemifields at different frequencies, ranging from roughly 2 Hz (transient responses) to 13 Hz (steady-state responses).

## Methods

### Participants

A total of 7 participants, 4 females and 3 males aged 22–28 years (median 25 years), took part in the study after providing informed consent. They reported no history of neurological or ophthalmological disorders. The study followed the tenets of the declaration of Helsinki and was approved by the local institutional review board. All participants had normal or corrected-to-normal vision (decVA > 1.0) as measured with the Freiburg Visual Acuity Test (FrACT) [30, 31].

### Stimuli and procedure

**Fig. 2:**
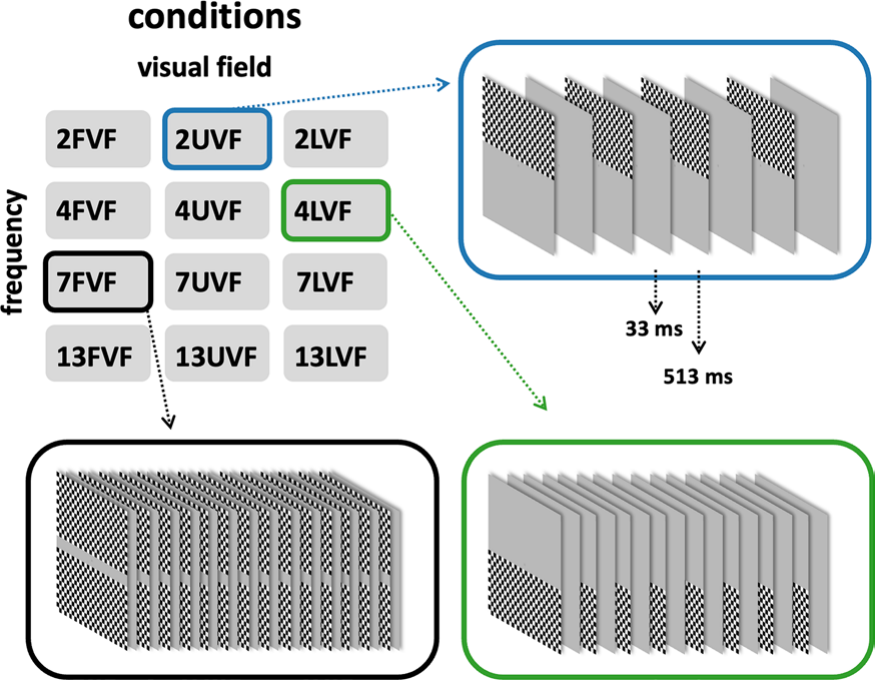
Paradigm design. Each trial comprised of a short sequence of checkerboard onset stimulation with a duration of about 2 s. Each single checkerboard stimulus was presented for 33 ms and was either constraint to the upper (UVF) or lower (LVF) visual field or extended over both hemifields (full visual field, FVF). During a stimulation block of approximately 5 min duration, stimulation sequences of different conditions (temporal frequencies and visual field regions) were randomly intermixed. Participants were instructed to fixate on a central fixation mark, and to report short luminance changes of this fixation mark. Top left: summary of possible conditions, with the number indicating the stimulation frequency and the capital letters indicating the stimulated visual field. Top right and bottom: exemplary trials. During inter-stimulus intervals and in between trials, the screen was homogenously gray

Stimuli were generated using PsychoPy [32, 33] for Mac and presented on a black and white CRT monitor (model 21CY9, Philips, Amsterdam, Netherlands) at a distance of 114 cm from the eyes of the participants. The stimuli consisted of an onset/offset checkerboard sequence that was displayed either in the upper visual field (UVF), lower visual field (LVF) or the full visual field (FVF), and either with an onset frequency of 1.8 Hz, 3.6 Hz, 6.9 Hz or 12.8 Hz. For simplicity, these frequencies will be labeled 2 Hz, 4 Hz, 7 Hz, and 13 Hz (Fig. 2). This resulted in twelve different experimental conditions (Fig. 2). Those are designated as 2UVF (2 Hz, upper visual field), 7FVF (7 Hz, full visual field), and so forth. The 96 × 96 individual elements of the checkerboard were of equal size (0.13° visual angle in both dimensions) independent of their position in the visual hemifield. In all conditions, a gray bar was located along the horizontal meridian (0.52° visual angle in vertical direction, see Fig. 2) in order to clearly separate the hemifields. The total size of the checkerboard was therefore 12.48° of visual angle horizontally and 13.00° of visual angle vertically. Michelson contrast of the patterns was 95%, and the mean luminance was 220 cd/m^2^. The gray screen during the inter-stimulus interval as well as the area surrounding the checkerboard also had this luminance.

A trial consisted of approximately 2 s of onset/offset checkerboard stimulation at a particular temporal frequency. Stimulus offset was 33 ms (3 frames at a framerate of 90 Hz) after stimulus onset, regardless of the stimulation frequency. The duration of the subsequent inter-stimulus interval depended on the stimulation frequency. Different stimulation frequencies had different numbers of stimulus appearances within one trial, namely 4 (~ 2 Hz), 8 (~ 4 Hz), 15 (~ 7 Hz), or 30 times (~ 13 Hz). Each condition was repeated 100 times, resulting in a total of 1,200 trials. The order of the conditions was randomized. Between trials, there were variable inter-trial-intervals with durations randomly drawn from a uniform distribution ranging from approximately 0.8–1.6 s. After a run of 100 trials (lasting about 5–6 min), the participants were able to take a break ad libitum.

In order to ensure central fixation and to reduce effects of varying spatial attention, the participants were instructed to steadily fixate a small centrally presented circle which changed its luminance for periods of 0.5 s at random times, on average once per trial (i.e., at a mean interval of approximately three seconds), but temporally uncorrelated to checkerboard stimuli. Participants responded to those luminance shifts by pressing buttons on a response box placed on their lap with either the left thumb (luminance decrement) or right thumb (increment).

### EEG recording

For EEG acquisition, we used a Brain Vision EEG system (Brain Products GmbH, Munich, Germany) with a sampling rate of 500 Hz and an FCz reference. Active electrodes were placed at 32 scalp positions according to the 10–10 system [34]. Scalp locations were cleaned using 70% isopropyl alcohol before the electrodes were positioned, and impedances were kept below 5 kΩ by using SuperVisc electrode gel (EASYCAP GmbH, Herrsching, Germany). All signals were band-passed at 0.1–70 Hz and saved to disk for offline analysis.

### EEG analysis

For analysis of the EEG-data, the MNE python package was used (version 0.19.2, http://martinos.org/mne) [35, 36]. Bad channels were rejected from further analyses (participant 6, channels P4 and O1 because of an impedance » 5 kΩ). Raw data were re-referenced to the average of TP9 and TP10 (mastoids), in accordance with [9, 14], and a low-pass filter with cutoff at 47.5 Hz was applied to remove mains interference. Eye blinks were detected at electrodes FP1 and FP2 using a threshold of 100 µV. Intervals of ± 250 ms around the time point of the peak amplitude of the blink artifact were assumed to be contaminated, and affected trials were discarded from further analyses. In most participants  « 10% of the trials had to be discarded due to eye blinks.

Analysis focused on electrodes Pz and Oz. Pz was chosen as polarity inversion was best seen there on a previous study [9]. We further analyzed electrode Oz, as it is often used in clinical diagnostics. However, we expected polarity inversion there to be weaker [9]. The raw EEG signal was divided into epochs starting from first stimulus onset of a trial and ending one full period after last stimulus onset within a trial. Baseline for the signal was chosen to be the 0.322 ms before each trial. Mean-centering but no linear detrending within an epoch was performed.

### Time domain analysis

Evoked responses were extracted by averaging the epochs of the respective conditions within participants. We obtained evoked responses both for individual participants, and as grand average for the full group. In addition to FVF, UVF, and LVF conditions, we computed a sum of UVF and LVF (SUM). The SUM signal corresponds to the hypothetical FVF stimulation, i.e., the sum of the single-field responses and it is compared to the actual full field response (FVF). A complete trial lasted about 2 s, depending on stimulation frequency. For the purpose of better visualization of details, only half of trial duration is depicted in Fig. 3.

**Figure:**
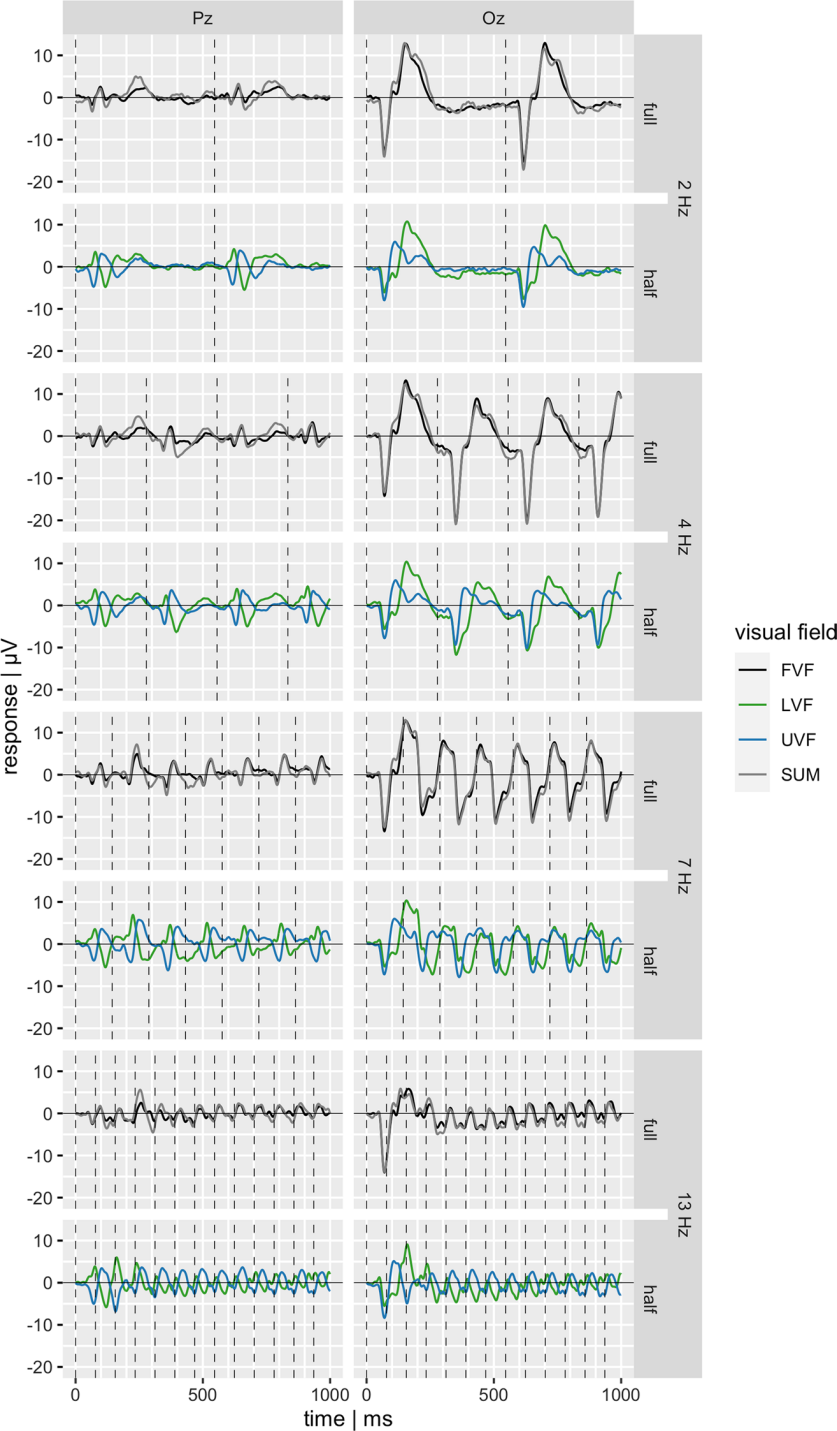

To quantify the relationship between LVF and UVF responses, we computed Pearson correlation coefficients between LVF and UVF responses for each stimulation frequency (2, 4, 7, and 13 Hz). We did this on a single participant level to account for inter-individual differences. For the transient conditions (2 and 4 Hz), we used an interval of 300 ms after the first stimulus onset of a trial to calculate correlation between UVF and LVF responses. For steady-state conditions (7 and 13 Hz), we discarded the first three periods to eliminate the transient responses at the beginning of a stimulation period and ensure that the system was in a steady-state equilibrium. We then used the full remaining trial to calculate the correlation coefficient. Correlation coefficients were then Fisher-R-to-Z transformed applying arctanh.

### Frequency domain analysis

A discrete Fourier transform was applied to steady-state responses (7 and 13 Hz) using the fast Fourier transform [37]. The ERP segments were first cut at the beginning (initial stimulus onset) and a full period after the last stimulus onset. From the resulting complex projection, we calculated the amplitude and phase angle at different frequency bins. This was done for all conditions (FVF, UVF, LVF, and SUM) in both steady-state stimulation frequencies (7 and 13 Hz). We additionally standardized the phase angles by subtracting the angle of the FVF response within a particular frequency from all responses within the respective frequency.

## Results

### Behavioral performance

Most participants were able to correctly indicate the luminance change of the fixation dot (mean hit rate 0.85 ± 0.18) within the short interval that it was presented. The mean reaction time of the correct responses was 0.43 s ± 0.07 s. One participant had slight difficulties in correctly identifying the luminance changes (hit rate of 0.48, see Table S1).

### Time domain

Evoked responses were calculated for all stimulation conditions across participants (Fig. 3). Both electrodes Pz and Oz were analyzed. For electrode Pz, a clear polarity inversion between LVF and UVF stimulation can already be seen in early time windows beginning at 60 ms (Fig. 3, left). In contrast, the Oz response shows opposite polarity mainly for a short time interval around 100–130 ms, but the shapes of the two curves differ for a longer time interval. Opposite polarity is present to some degree with all stimulation frequencies (Fig. 3, right). Differences between hemifields, but not necessarily a polarity inversion, can also be seen at later time intervals in both Pz and Oz responses. The sum of the signals of the UVF and LVF conditions (SUM) closely matches the FVF response in all stimulation conditions and at all electrodes (Fig. 3). Notably, at electrode Pz, polarity inversion seems to result in rather destructive interference between LVF and UVF transient responses, leading to a smaller absolute FVF and SUM response. On the contrary, at electrode Oz, inference appears to be constructive. Both constructive and destructive effects get weaker with increasing stimulation frequency. Figures S1–S7 illustrate the evoked responses on a single-participant level. Similar effects can be observed than with the grand average across participants. However, the graphs illustrate a substantial inter-individual variability in the shape of the response. Whereas in some participants early responses to the stimuli in UVF and LVF seem inverted in polarity, in others it looks rather like a phase shift in certain components.

**Fig. 4:**
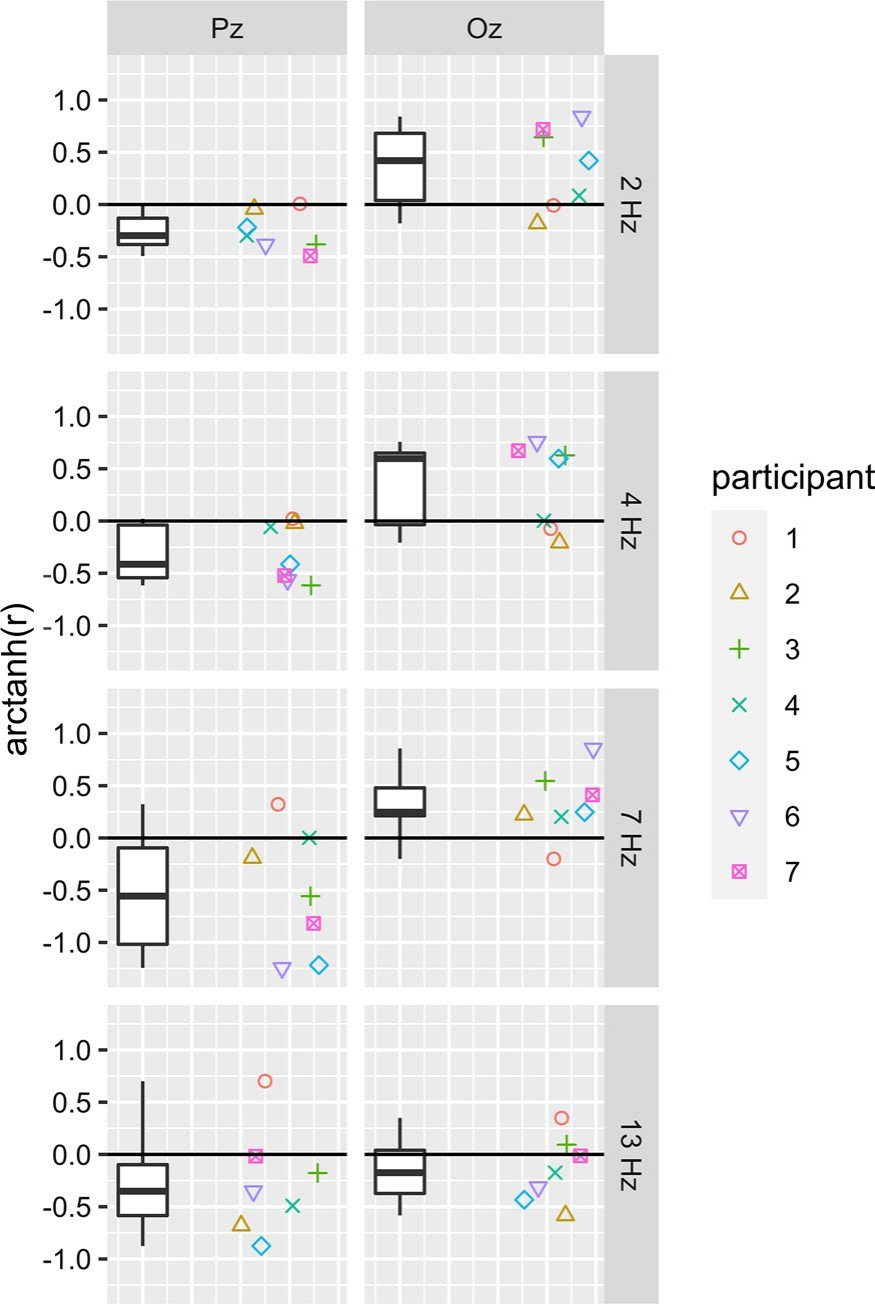
Correlation between LVF and UVF time course. Displayed are individual participants’ Pearson correlation coefficients (Fisher-R-to-Z transformed) for each stimulation condition from top to bottom (2, 4, 7, and 13 Hz) for electrode Pz (left column) and Oz (right column). The boxplots illustrate the distribution of the coefficients (0.0, 0.25, 0.5, 0.75, and 1.0 quantiles). The individual participants (Fisher-R-to-Z transformed, a.k.a. arctanh transformed) correlation coefficients are illustrated next to the boxplots. Negative correlations indicate opposite time courses, which in turn indicate polarity inversion. For electrode Pz, correlation coefficients were predominantly negative throughout all stimulation frequencies, indicating frequency-independent polarity inversion. Notable is the high inter-individual variability. In electrode Oz, mostly positive correlations can be seen in the transient conditions. However, correlation coefficients became predominantly negative in 13 Hz condition at electrode Oz

Furthermore, we calculated the Pearson correlation (Fisher-R-to-Z-transformed) of the LVF and UVF time courses for each individual participant (Fig. 4). A negative correlation is indicative of a polarity inversion. Figure 4 illustrates high variability between participants in the correlation coefficients. However, the single participant patterns closely agreed with the group pattern seen before. Namely, at all stimulation frequencies, a polarity inversion could be seen in Pz. In contrast, consistent polarity inversion at Oz occurred only at the highest stimulation frequency (13 Hz). Although the group size is not sufficient for a detailed assessment of sex effects, inspection of single-participant data did not reveal any systematic differences.

### Frequency domain

**Fig. 5:**
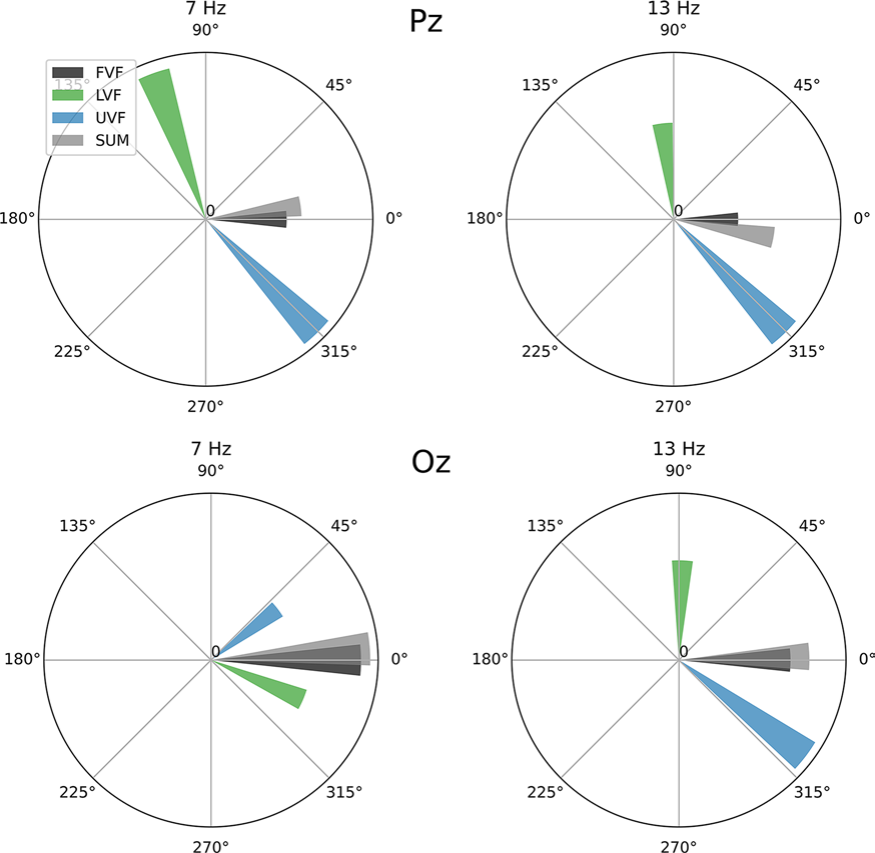
Phase shift and amplitude of the steady-state stimulations (7 and 13 Hz) as obtained by Fourier transformation of the grand means. Polar plots show the absolute electrophysiological responses in the stimulation frequency in µV (magnitude of each bar in radial direction), and the phase shift (azimuth) compared to the FVF stimulation (0°). Upper row: electrode Pz, lower row: electrode Oz. Left column: stimulation at 7 Hz, right column: stimulation at 13 Hz. Importantly, for different temporal frequencies, the same angular phase difference corresponds to different time shifts

Steady-state VEPs are often analyzed in the frequency domain. We therefore also illustrated the signals’ phase diagrams for the first harmonic (Fig. 5). All steady-state stimulation conditions (7 & 13 Hz) elicited strong responses at the respective stimulation frequencies. For all conditions and at both electrodes, a phase shift between UVF and LVF can be seen. In three out of four cases, the shift is quite pronounced, and between 135° and 180°. In one case (7 Hz at electrode Oz), phase shift is just below 90°. This closely aligns with the insights from Figs. 3 and 4, demonstrating only a moderate phase shift rather than a full phase shift which would indicate polarity inversion at the respective frequency bin. Notably, the amplitudes of the FVF and SUM are weaker than the half field stimulations at Pz, indicating a destructive interaction between components. In contrast, constructive interactions can be seen in electrode Oz at 7 Hz, and neither constructive nor destructive interactions are seen at 13 Hz, closely agreeing with the insights from Fig. 3.

## Discussion

In the present study, we assessed visual evoked potentials to stimulation of the upper and lower hemifield and to the combined stimulation of both hemifields at temporal frequencies ranging from 2 to 13 Hz. Several findings are of particular interest with respect to the polarity of responses.

1. At Pz, polarity inversion can be observed in the time course data at all temporal frequencies for at least some components (Fig. 3 & 4).
2. Oz time course data only show inversion during short time intervals. Inversion appears to be the dominant mode at 13 Hz (Fig. 3 & 4).
3. The first harmonic of the steady-state responses (7 & 13 Hz) shows a phase shift between hemifields which is consistent with an inversion (Fig. 5).
4. The sum of the hemifield responses typically matches the full field response quite well (Fig. 3 & 5).

The present findings thus reveal a complex pattern of partial polarity inversion in steady-state VEPs when switching stimulation between upper and lower hemifields. It is particularly interesting that opposite polarity is rather pronounced with high stimulation frequencies. The interaction between electrode (Pz vs. Oz) and stimulation frequency (7 Hz vs. 13 Hz) suggests that different components, originating from different cortical sources, superimpose to varying degrees in the steady-state response and are differentially affected by inversion. The stimulation frequency has an important role as it may alter the shape of the response to an individual pattern appearance within the stimulus sequence and also affects how components with different temporal characteristics may interact constructively and destructively [28]. In particular, those response components that occur relatively late in the transient VEP may not manifest well at high stimulation rates as these components extend over a much longer time interval than the inter-stimulus interval. In other words, the fact that polarity inversion manifests in particular at a high stimulation rate might be evidence of the relative contribution of early visual areas to the steady-state VEP being largest at high stimulation frequencies.

While a phase difference of around 180° is expected for a polarity inversion, an interpretation of phase differences as literally representing a shift of the phase appears inappropriate. This becomes particularly obvious when looking at the 7 Hz Oz response. The traces of the upper and lower visual field cannot be translated into one another by shifting them in time. Rather, the differences in curve shape are the main determinant of the phase differences. This could be caused, for example, by some response components reversing and others keeping their polarity. Contributions to the effect could also come from differences in processing characteristics between the two hemifields, possibly depending on the specific stimulus [38, 39].

In the light of the cruciform model, our results closely align with the idea that the horizontal meridian would (on average) be located on the ventral flank of the cruciform (Fig. 1). For instance, the LVF stimulation was more similar to the FVF than the UVF stimulation was (Fig. 3). That location might, however, vary between participants [40]. Further, a certain degree of interindividual variability, as evident from Fig. 4 and Figures S1–S7, is not surprising given considerable interindividual differences in general cortical folding.

An inversion has important practical consequences, as it reduces the response that is recorded to full-field stimulation. In such cases, steady-state responses could possibly be boosted by introducing a counteracting phase shift between the stimulus sequences in the upper and lower hemifields (possibly further refined by accounting for differences between eccentricities). This is of particular interest when the VEP is used to determine sensory thresholds, such as visual acuity, where an improved signal-to-noise ratio near threshold might make the threshold estimates more reliable. A potential caveat for such applications concerns the effect of check size and type of visual impairment on the response shape, which might result in different patterns of constructive or destructive interference between sequential responses to individual pattern onsets [28]. Importantly, however, the VEP-based threshold estimation typically relies on the presence of a response versus its absence. Because the shape of the response is normally not evaluated in such applications, it is in principle a valid approach to try out different counteracting phase shifts or other data operations for each tested individual and take the one that yields the best signal-to-noise ratio. However, any practical implementation would need to avoid creating a spurious response from aligned noise.

In summary, the present study reveals that the impact of the inversion effects in steady-state responses partly depends on the stimulation frequency, possibly due to the relative contribution of different cortical areas to the measured steady-state VEP. It opens up ways to enhance responses by accounting for the phase shift between hemifields.

## Supplementary Information

Below is the link to the electronic supplementary material.

Supplementary file1 (PNG 853 KB)

Supplementary file2 (PNG 826 KB)

Supplementary file3 (PNG 686 KB)

Supplementary file4 (PNG 705 KB)

Supplementary file5 (PNG 793 KB)

Supplementary file6 (PNG 681 KB)

Supplementary file7 (PNG 726 KB)

Supplementary file8 (PNG 866 KB)

Supplementary file1 (DOCX 28 KB)

## Data Availability

Data are available from the corresponding author upon reasonable request.

## Declarations

### Statement on the welfare of animals

This article does not contain any studies with animals performed by any of the authors.
