# Correction to: Temporal frequency dependence of the polarity inversion between upper and lower visual field in the pattern-onset steady-state visual evoked potential

**DOI:** 10.1007/s10633-022-09913-8

**Published:** 2023-01-28

**Authors:** Roman Kessler, Sven P. Heinrich

**Affiliations:** 1 Department of Neuropsychology, Max Planck Institute for Human Cognitive and Brain Sciences, Leipzig, Germany; 2 Eye Center, Faculty of Medicine, Medical Center – University of Freiburg, Freiburg, Germany

**Correction to: Doc Ophthalmol**

https://doi.org/10.1007/s10633-022-09904-9x

In the original article, the formatting of “<<” and “>>” was indicated with different symbols.

For example,
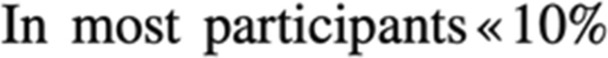


and,
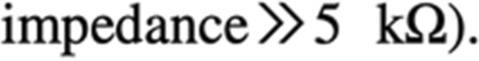


Unified symbols are used to indicate greater than and less than.

The original article has been corrected.

