# Supplementary figures and images for: Temporal frequency dependence of the polarity inversion between upper and lower visual field in the pattern-onset steady-state visual evoked potential
Source: Doc Ophthalmol. 2022 Oct 22;146(1):53–63. doi: 10.1007/s10633-022-09904-9 (PMC9911476; doi:10.1007/s10633-022-09904-9)

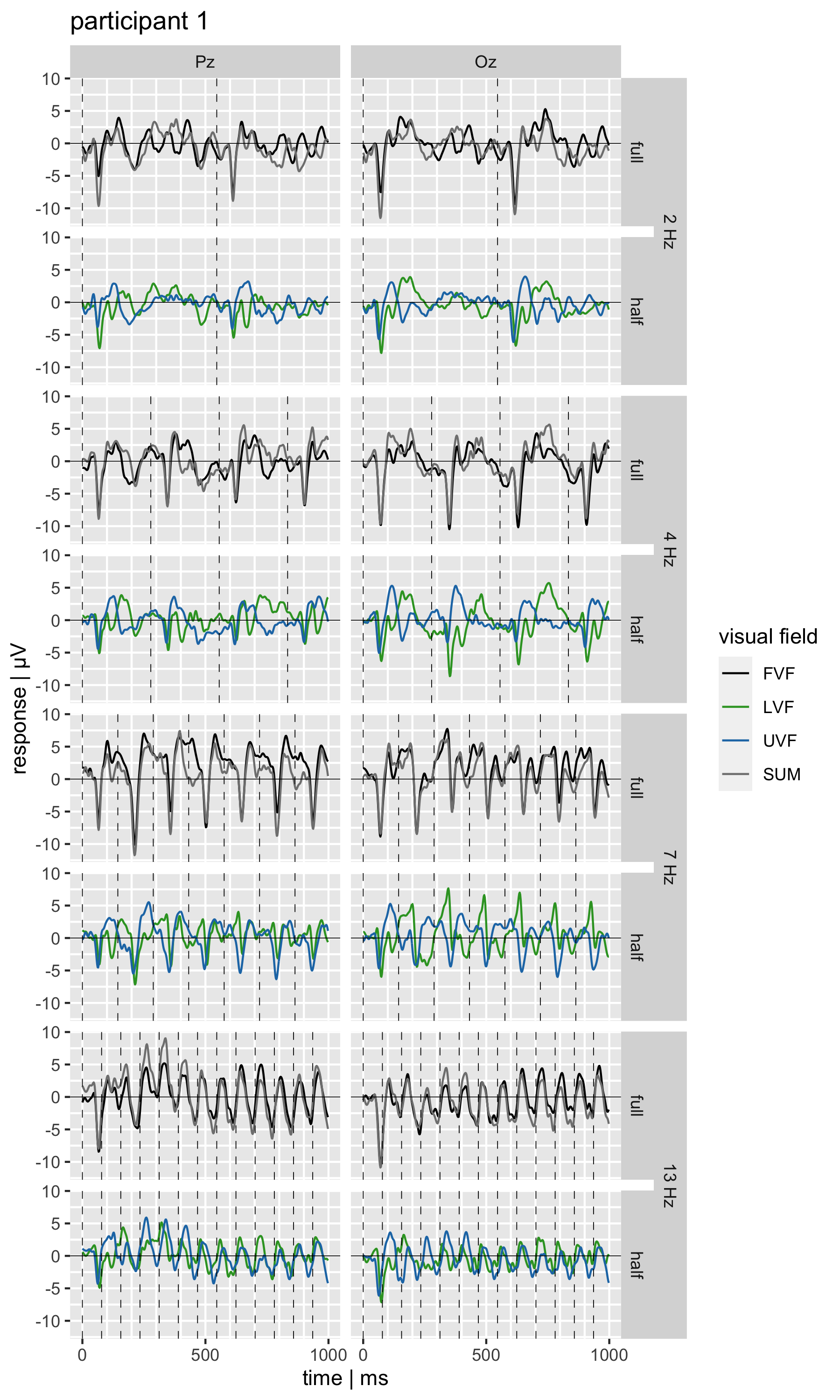

Supplement: Supplementary file 1 — Supplementary file1 (PNG 853 KB) [file 10633_2022_9904_MOESM1_ESM.png]

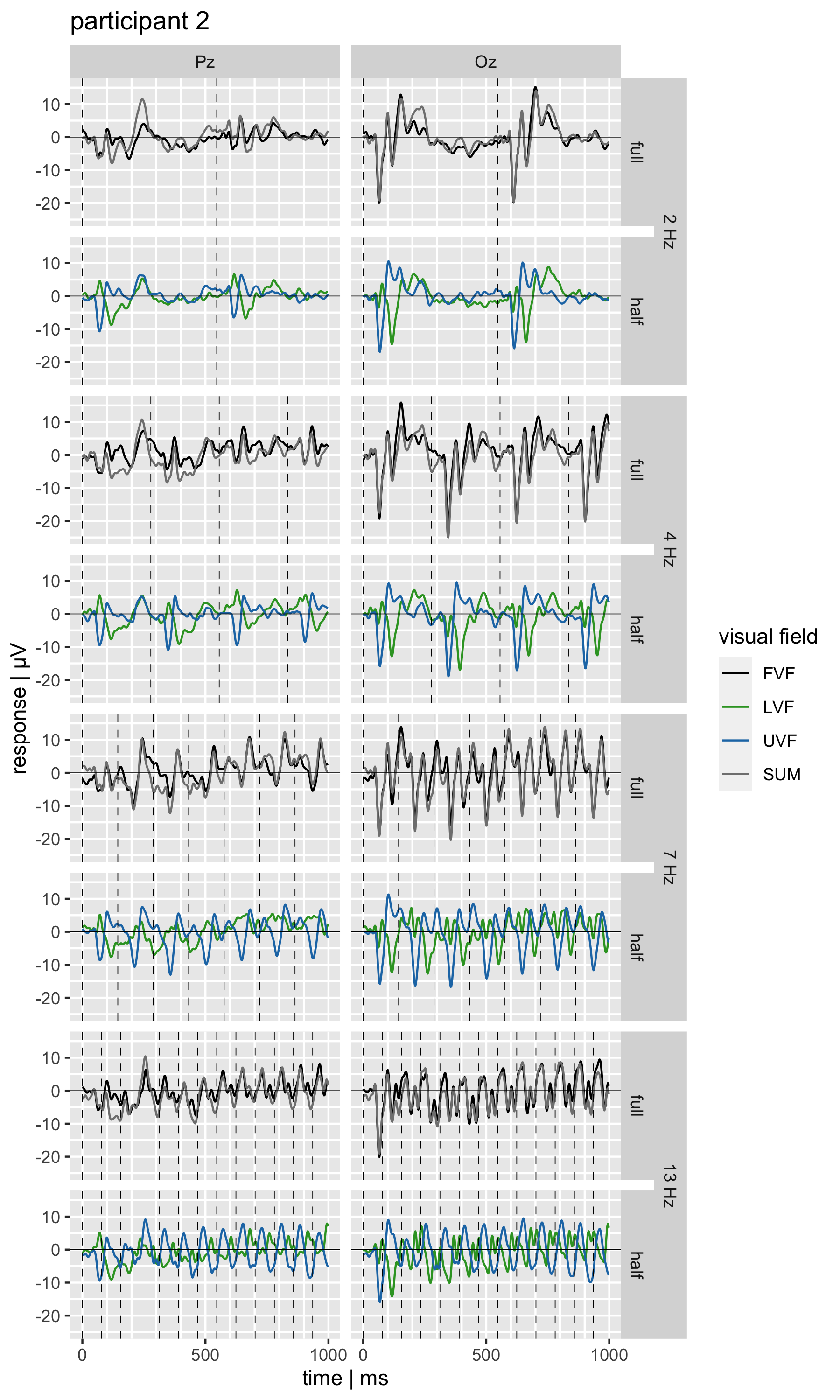

Supplement: Supplementary file 2 — Supplementary file2 (PNG 826 KB) [file 10633_2022_9904_MOESM2_ESM.png]

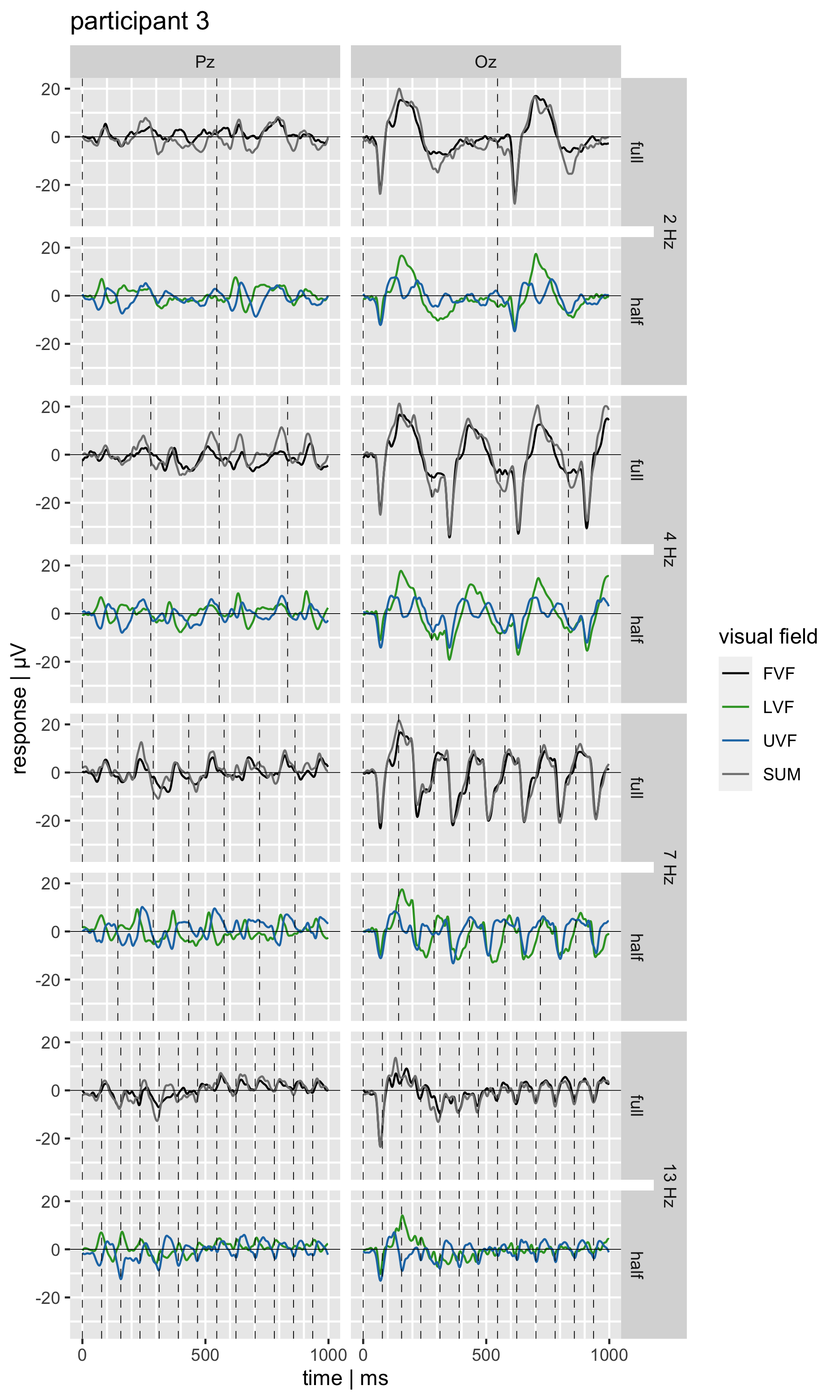

Supplement: Supplementary file 3 — Supplementary file3 (PNG 686 KB) [file 10633_2022_9904_MOESM3_ESM.png]

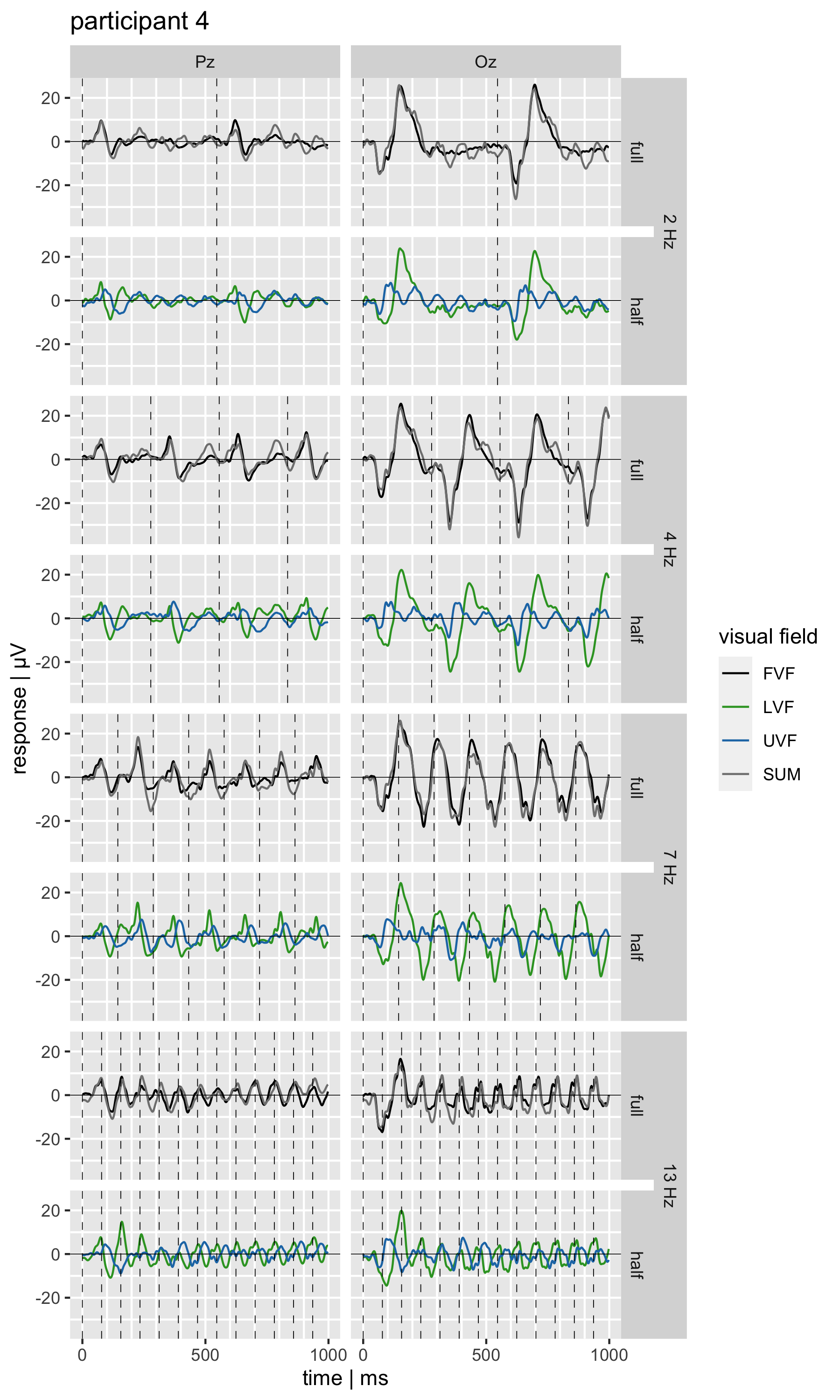

Supplement: Supplementary file 4 — Supplementary file4 (PNG 705 KB) [file 10633_2022_9904_MOESM4_ESM.png]

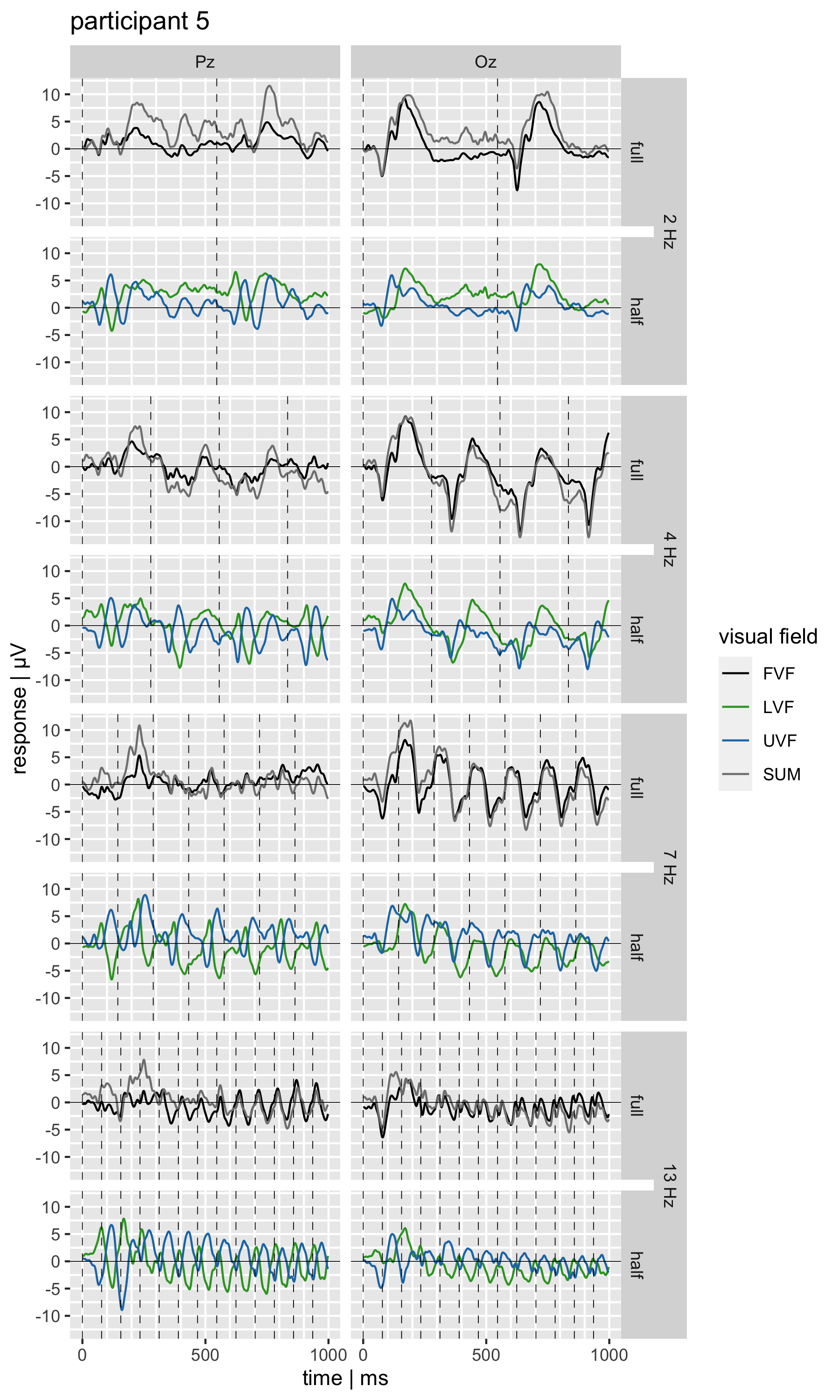

Supplement: Supplementary file 5 — Supplementary file5 (PNG 793 KB) [file 10633_2022_9904_MOESM5_ESM.png]

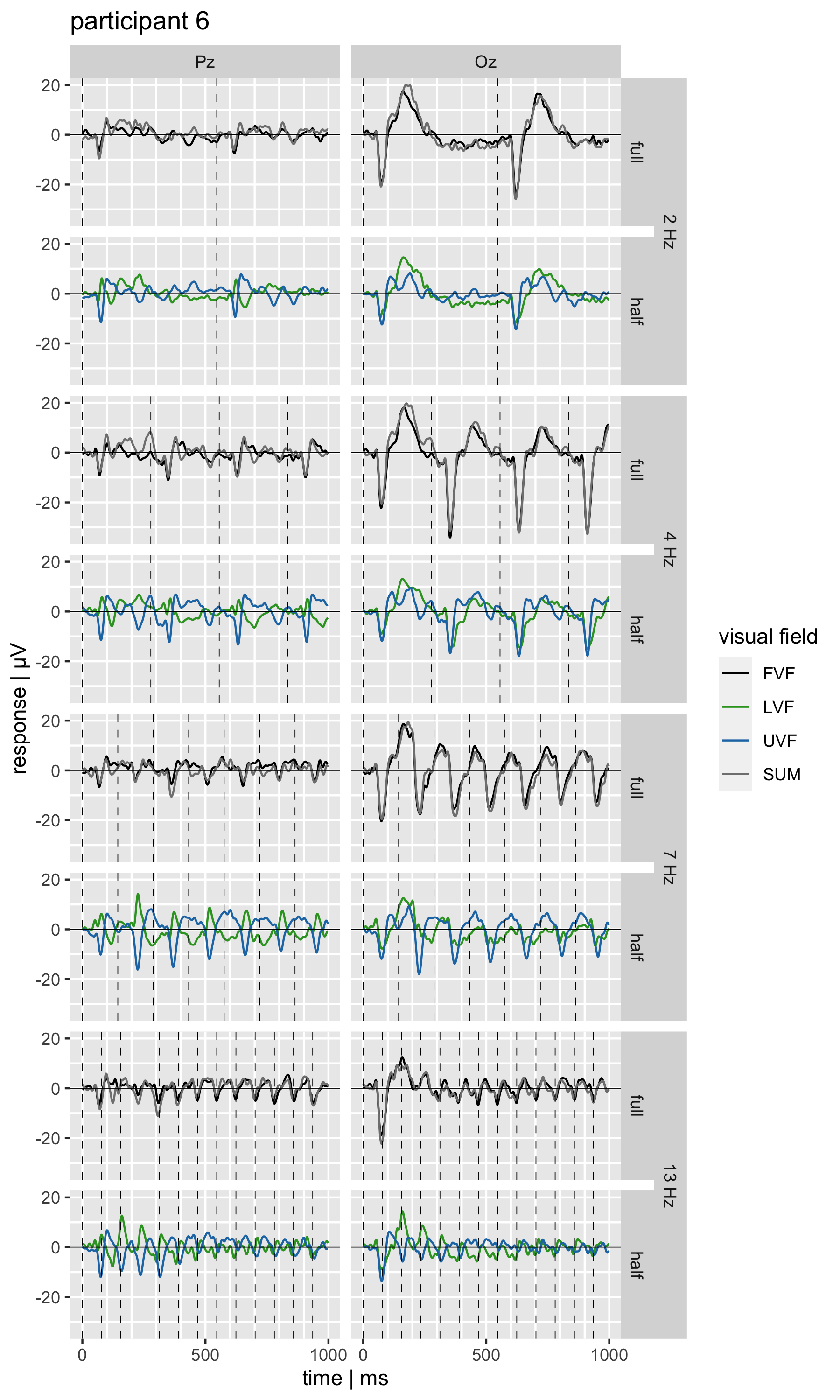

Supplement: Supplementary file 6 — Supplementary file6 (PNG 681 KB) [file 10633_2022_9904_MOESM6_ESM.png]

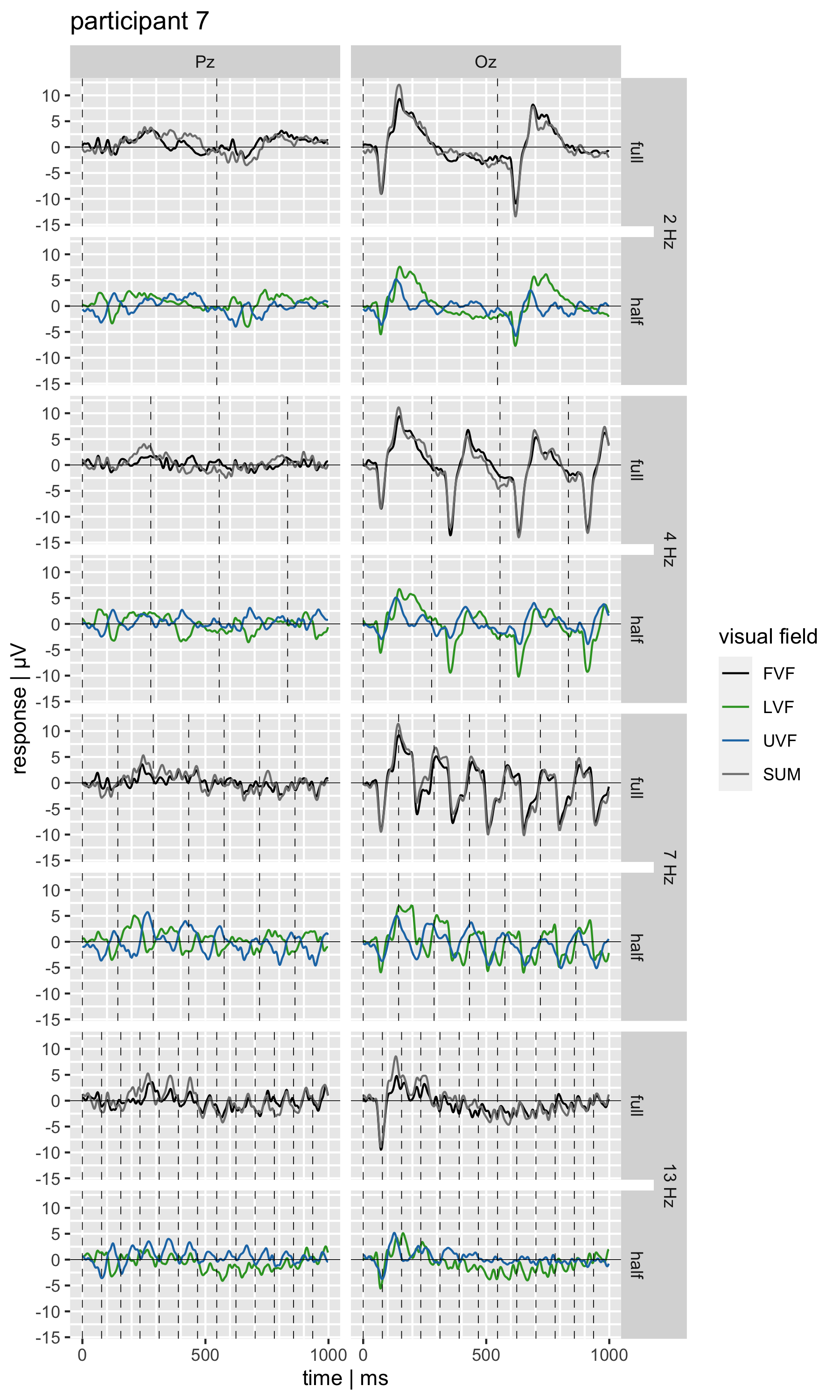

Supplement: Supplementary file 7 — Supplementary file7 (PNG 726 KB) [file 10633_2022_9904_MOESM7_ESM.png]

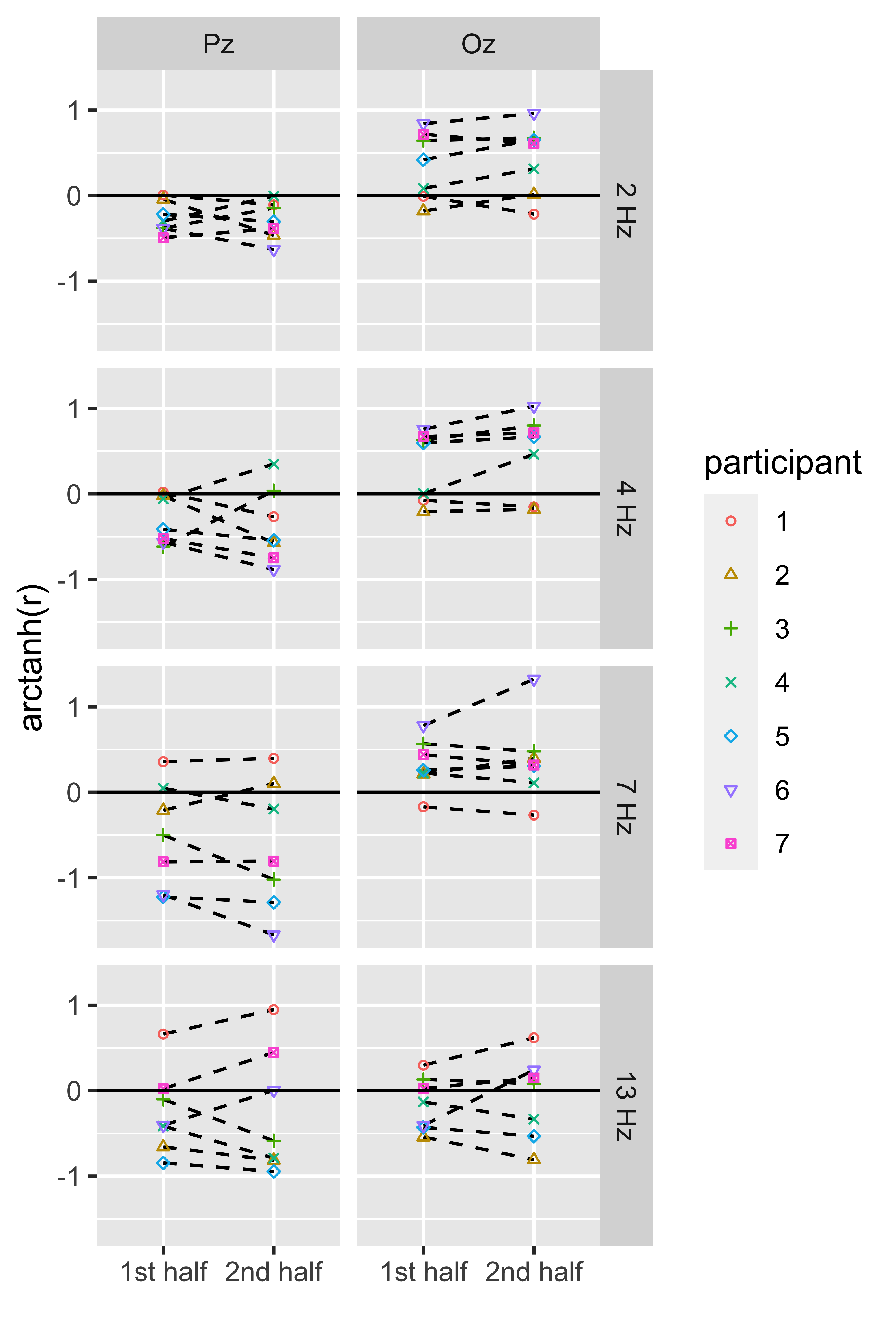

Supplement: Supplementary file 8 — Supplementary file8 (PNG 866 KB) [file 10633_2022_9904_MOESM8_ESM.png]
