# Supplementary material for: Temporal frequency dependence of the polarity inversion between upper and lower visual field in the pattern-onset steady-state visual evoked potential
Source: Doc Ophthalmol. 2022 Oct 22;146(1):53–63. doi: 10.1007/s10633-022-09904-9 (PMC9911476; doi:10.1007/s10633-022-09904-9)
Supplement: Supplementary file 9 — Supplementary file1 (DOCX 28 KB) [file 10633_2022_9904_MOESM9_ESM.docx]

# Supplements

## Participant information

***Table S1:*** *Participants’ demographics, visual acuity and performance in detection of the fixation marks’ luminance shifts.*

| participant | sex | Age [years] | visual acuity (decimal) | hit rate | reaction time [s] |
| --- | --- | --- | --- | --- | --- |
| 1 | m | 27 | 1.67 | 0.98 | 0.34 |
| 2 | f | 28 | 2.00 | 0.99 | 0.35 |
| 3 | f | 25 | 1.50 | 0.90 | 0.42 |
| 4 | f | 22 | 1.49 | 0.48 | 0.51 |
| 5 | m | 28 | 2.00 | 0.88 | 0.44 |
| 6 | f | 25 | 1.45 | 0.78 | 0.52 |
| 7 | m | 22 | 1.14 | 0.91 | 0.42 |
| mean |  |  |  | 0.85 | 0.43 |
| median |  | 25 | 1.50 | 0.90 | 0.42 |

## Single Participant evoked responses

To further demonstrate the individual variability in evoked responses across participants, the individual time series are displayed for each participant and stimulation condition (Figures S1 to S7). The average across participants is summarized in Figure 3 within the main manuscript.
